# Tunnel wash water in a cold climate: characteristics, ecotoxicological risk, and effect of sedimentation

**DOI:** 10.1007/s11356-024-35773-7

**Published:** 2025-01-06

**Authors:** Nadine A. Sossalla, Wolfgang Uhl, Hanne Vistnes, Subhash Srikantha Rathnaweera, Eilen Arctander Vik, Thomas Meyn

**Affiliations:** 1 Department of Civil and Environmental Engineering, Norwegian University of Science and Technology, S. P. Andersens Veg 5, 7031 Trondheim, Norway; 2 Aquateam COWI AS, Karvesvingen 2, 0579 Oslo, Norway; 3 COWI AS, Karvesvingen 2, 0579 Oslo, Norway

**Keywords:** Tunnel wash water, Polycyclic aromatic hydrocarbons, Sedimentation, Environmental quality standards

## Abstract

The characterization of tunnel wash water (TWW) from 12 Norwegian tunnels showed very high concentrations of total suspended solids (TSS), metals, and polycyclic aromatic hydrocarbons (PAHs). Iron (Fe), aluminum (Al), and manganese (Mn) were mainly particle-associated. They are efficiently removed by sedimentation, while the dissolved concentrations of toxic metals like Cu, Zn, and As did not change. Polycyclic aromatic hydrocarbon (PAH) concentration was higher in the dissolved than in the particulate fraction. Sedimentation treatment reduced the particulate share by about 50%. In contrast to metals, the dissolved fraction decreased by about 90% during sedimentation, most probably due to adsorption to particles that are subsequently removed during the process. The concentration levels of 8 out of 16 PAHs, Cu, Zn, and As resulted in a water quality classification of poor or very poor. Acute toxic effects could be expected after short-term exposition. After sedimentation, this was the case for only two PAHs because more harmful compounds with three to six rings were preferably removed. These may adsorb better to particles than PAHs with two rings. No change was observed for the metals. A closer evaluation of secondary treatments of TWW, including conventional technology and mobile solutions, is therefore recommended. Finally, indications for a seasonal pollution variation have been seen, mainly related to the use of salt as a de-icing agent in road maintenance.

**Graphical Abstract:**

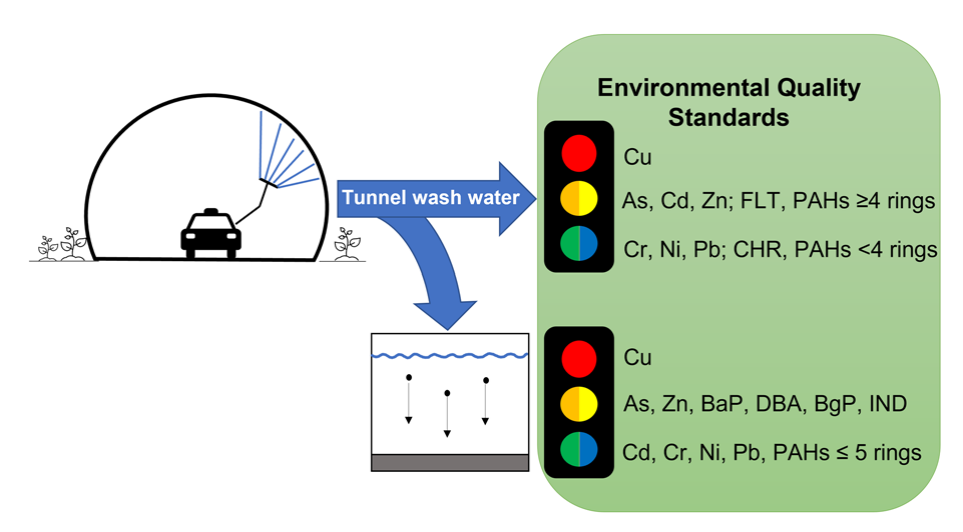

**Supplementary Information:**

The online version contains supplementary material available at 10.1007/s11356-024-35773-7.

## Introduction

A thorough and well-designed road infrastructure is essential for transportation and travel in a modern civilization. In addition to proper roads, in some regions, tunnels are an indispensable part of the urban infrastructure (Petersen et al. 2016). To reduce costs for transportation and improve road users’ safety, and due to the demanding mountainous landscape with fjords, Norwegian road infrastructure includes more than 1000 tunnels with a length of over 800 km (Meland et al. 2010a). On roads, pollutants are relocated by wind and rain, which can cause environmental harm in the receiving waters (Crabtree et al. 2008). However, compared to open roads, the environmental conditions in tunnels are considerably different. In tunnels, pollutants, such as dust, dirt, road salt, particles from abrasion of the road surface and tyres, and combustion residues from vehicles, accumulate on the pavements, walls, and traffic signs over time. For safety and maintenance reasons, tunnels are washed on a regular basis (2–12 times per year) (Chen et al. 2023; Paruch and Roseth 2008b). The washing routine depends on factors such as tunnel size and traffic load (Meland and Rødland 2018). Furthermore, the washing procedure itself varies, depending on the amount of pollution. During a *technical wash*, the traffic signs and technical gear are washed, whereas once or twice a year a so-called *full wash* is performed, and the entire surface of the tunnel, including all installations, is cleaned (Petersen et al. 2016). In some tunnels, detergents are used in addition to better cleaning performance (Norwegian Public Roads Administration 2014). The water generated during a tunnel wash is known as tunnel wash water (TWW).

The origin of the pollutants is related to vehicular traffic, i.e. the amount of traffic, types of vehicles, and way of operation (Borris et al. 2016). Road runoff in general is known to contain high levels of trace metals, organic pollutants (e.g. polycyclic aromatic hydrocarbons), and particles (e.g. microplastics or particles from construction material), amongst others (Flanagan et al. 2019; Hilliges et al. 2013). As these contaminants accumulate in the tunnels, TWW generally contains the same types of contaminants as road runoff but in elevated concentrations (Meland 2012b; Paruch and Roseth 2008b). Metals are released from different parts of the vehicles, such as tyres (lead, zinc), exhaust gases (cobalt, nickel), brake linings (antimony, copper, iron, lead, zinc), batteries (zinc), and engine wear and welded metal plating (chrome, nickel) (Huber et al. 2016; Meland et al. 2010a; Müller et al. 2020). In addition to vehicles, road components contribute to the release of pollutants. Road paints used for safety fences contain lead and zinc (Huber et al. 2016). Organic pollutants, such as polycyclic aromatic hydrocarbons (PAHs), are released by vehicle wear, during vehicle operation, and through road abrasion and abrasion by tyres (Huber et al. 2016).

For road runoff and with an annual average daily traffic (ADT) of more than 3000 vehicles per day, sedimentation is required as a first treatment step, while for an ADT > 15,000 vehicles per day, a secondary treatment step must be implemented (Statens vegvesen 2022a). Previous studies concluded that untreated TWW causes ecotoxicological harm to the environment (Meland et al. 2010a; Petersen et al. 2016). Therefore, the collection and treatment of TWW are recommended if the water recipients are highly vulnerable (Statens vegvesen 2022c). Based on the European Water Framework Directive (EU WFD), which requires good ecological standards for water bodies, the Norwegian Environment Agency (Miljødirektoratet) has implemented the status classes for water bodies in the national law (Miljødirektoratet 2020) and established regional water management plans and action programmes to reach good water quality conditions, at least Class II (Miljødirektoratet 2021).

Nowadays, most new tunnels are built with a sedimentation basin for TWW treatment. A sedimentation period can last between 14 and 35 days. Furthermore, if a detergent is used in the washing process, it must be degradable within 28 days by 60% (The European Commission 2004). It should be noted that this regulation provides for testing at room temperature (19–24 °C), which differs from the real conditions in a sedimentation basin in the tunnel, where cooler temperatures normally prevail. In Norway, the sedimentation-treated TWW is released into a water recipient. If TWW is discharged to the sewer system, municipal requirements for industrial wastewater must be met.

Previous studies focused on dissolved compounds (Hallberg et al. 2014; Meland and Rødland 2018; Stotz and Holldorb 2008). However, for a holistic understanding of the challenges resulting from TWW quality, both particulate and dissolved fractions must be considered. Also, for the design of effective and cost-efficient treatment processes, knowledge about the distribution of particle-bound and dissolved contaminants is of utmost importance.

Metals and PAHs, as they have been measured in TWW, may induce environmental harm. Metals such as Al, Fe, Cu, and Pb have been found to be toxic to fish, accumulating in their gills, and causing disruption of ion regulation and alteration of blood glucose levels, as well as triggering an oxidative stress response (Laurén and McDonald 1985; Meland et al. 2010b). For PAHs, fluoranthene (FLT) and pyrene (PYR) have been observed in embryo-larval bioassays to cause acute effects on early development (Bellas et al. 2008), while benzo[g,h,i]perylene (BgP) and indeno[1,2,3-c,d]pyrene (IND) are possible carcinogens and therefore problematic for the environment (Srogi 2007). Combined exposure to metals and PAHs has been reported to result in unexpected co-toxic effects. Gauthier et al. (2014) concluded a more-than-additive mortality. In contrast to mixtures with additive effects, mixtures with more-than-additive effects have a higher overall toxicity than the sum of the individual pollutants. In particular, Gauthier et al. (2014) summarized that the effects on mechanisms, such as the disruption of ion regulation, the imbalance of reactive oxygen species, and the damage to the cell membrane by PAHs, which can lead to higher intracellular metal concentrations, point in this direction. In addition, the detoxification processes of metals or PAHs may be negatively affected in the presence of the other pollutant group.

Consequently, the objectives of this study were to provide an understanding of the concentrations of contaminants in TWW in the particulate and dissolved fractions and to further evaluate the effect of sedimentation as it is currently practised. Finally, an evaluation of ecotoxicological risk from fresh and sedimentation-treated TWW was conducted.

## Materials and methods

### Concept of the study

TWW quality data that were mainly gathered from samples collected during a 3-year period (2019–2022) from 12 tunnels were evaluated for different types of washes, seasons, and in some cases after several days of sedimentation as post-treatment. The water quality of the fresh and sedimentation-treated TWW is compared to the applicable environmental quality standards (EQS), and the ecotoxicological risk is derived.

In addition, the treatment effectivity of the sedimentation process and the water quality of the treated TWW were investigated. The evaluation before and after sedimentation will support recommendations for future treatment designs.

### Sampling sites and methods

TWW was sampled from 12 tunnels in Norway, specifically in the area surrounding the capital of Oslo, and in the urban area of Trondheim, in September 2014, and between May 2019 and March 2022. The tunnel lengths are between 130 and 3867 m, with up to two lanes per tube, and the annual ADT was in the range of 9744–46,945,000 in 2022 (Table 1). Most tunnels have been rock-blasted, and their walls are covered with concrete elements and shotcrete at the ceiling. Smestad tunnel (SmT) has been built by concrete casting in an open construction pit. The climate in the whole sampling region can be characterized as oceanic climate (Cfb), and the average temperature ranges from − 3 °C in January to 18 °C in July. Precipitation is varying from around 50 mm per month in January to 105 mm in August (Norwegian Meteorological Institute). Further information is summarized in Supplementary Information B, Table B1.

**Table 1:** Tunnel length, number of tubes and lanes, annual average daily traffic (ADT), and the volume of the sedimentation basin of the investigated tunnels. The data for ADT were available for the year 2022 (Statens vegvesen 2022b)

Tunnel	Tunnel length (m)	Numbers of tubes/lanes per tube	Annual average daily traffic (ADT)	Volume of sedimentation basin (m^3^)
Bamble (BaT)	760	1/1	17,121	10
Bjørnegård (BjT)	2300	2/2	31,091	400
Fløyheia (FlT)	510	2/2	10,400	145
Granfoss (GaT)	2300	2/2	30,100	360
Grillstad (GrT)	750	2/2	34,632	96
Hesthag (HhT)	620	2/2	10,200	164
Nordby (NT)	3867	2/2	39,500	174
Smestad (SmT)	494	2/2	44,000	93
Strindheim (SrT)	2600	2/2	23,757	40
Torsbuåsen (TbT)	700	2/1&2	12,000	280
Træfjell (TfT)	130	2/2	10,200	139
Tåsen (TaT)	1350	2/2	46,945	194

Tunnel washes usually took place during the night. It must be noted that washing procedures are not standardized completely and can vary. Generally, a sweeper removes all the coarse dirt from the street. Then, all surfaces are washed using drinking water. Sometimes, but not always, a detergent is added to the wash water. The wash can be done with high- or low-pressure using noodles and/or brushes. After washing, the road is usually vacuumed by a sweeper to remove the remaining dust and wash water (Meland 2012b). The TWW is collected in a drainage system and finally in a sedimentation basin. As far as we know, none of the studied sedimentation basins were cleaned additionally between washes. Therefore, sedimented particles from several previous washes might have been resuspended when the ongoing wash water was pumped into the sedimentation basin.

Samples of fresh TWW were collected from the sedimentation basins early in the mornings after the washes. Due to the conditions on-site, standardized sampling was impossible. Samples were taken directly from the upper part of the sedimentation basins using buckets or submersible pumps in the following tunnels: Bamble (BaT), Bjørnegård (BjT), Fløyheia (FlT), Granfoss (GaT), Grillstad (GrT), Hesthag (HhT), Nordby (NT), Smestad (SmT), Torsbuåsen (TbT), Tærfjell (TfT), and Tåsen (TaT). Samples from BjT, GrT, SmT, and TaT were analyzed in the laboratories of the Norwegian University of Science and Technology (NTNU), Trondheim. Samples from all other tunnels were analyzed at commercial laboratories.

### Sedimentation conditions

The effect of sedimentation was studied on-site under field conditions. For samples of sedimentation-treated TWW, field sampling was done at eight tunnels (BaT, BjT, FlT, GrT, HhT, SmT, TbT, TfT), before the water was discharged. The time between the wash and the sampling varied between 7 and 44 days, depending on the tunnel. The sedimentation process for BaT was investigated more thoroughly, and various samples were taken after the wash and before the water discharge (SI_B7).

### Water quality analysis

Common water quality parameters like pH, electrical conductivity (EC), turbidity (TUR), total solids (TS) and total suspended solids (TSS; NS-EN 872:2005), and dissolved organic carbon (DOC, TOC Apollo 9000; Tekmar, Mason, OH, U.S.A.) were analyzed.

Regarding metals, based on the results of preliminary screening, the samples were analyzed for aluminium (Al), arsenic (As), copper (Cu), cadmium (Cd), chromium (Cr), iron (Fe), lead (Pb), mercury (Hg), nickel (Ni), and zinc (Zn), as well as the sum of EPA 16 PAHs (Σ_16_PAH).

The methods for metal and PAH analysis provided at NTNU are summarized in Supplementary Information A, Section A1.2. Briefly, total metal concentrations were obtained after the digestion of the unfiltered sample. Metals were analyzed by inductively coupled plasma mass spectrometry.

The metal concentration in the particulate fraction (*C*_part_) was calculated according to Eq. 1 from the total (*C*_tot_) and the dissolved (*C*_diss_) metal concentration in µg/L.

1 $${C\left(\text{Me}\right)}_{\text{part}}=C{\left(\text{Me}\right)}_{\text{tot}}-C{\left(\text{Me}\right)}_{\text{diss}}$$

However, for PAHs, the concentration in the particulate fraction (> 0.45 µm) was obtained after accelerated solvent extraction of the particles collected on the membrane filter, while the dissolved phase PAH fraction (< 0.45 µm) was extracted from the filtered samples by solid-phase extraction. The extracts obtained were analyzed by gas chromatography–mass spectrometry (GC–MS). The concentrations for dissolved and particulate fractions were obtained by normalizing the results from GC–MS measurement to the sample volume. A detailed description of the method is provided by Vistnes et al. (2022).

### Data handling and statistical analysis

The removal effectivity of the sedimentation process, in %, was calculated according to Eq. 2 from the initial concentration (*C*_init_) and the concentration after the respective sedimentation time (*C*_sed_).

2 $$\text{Removal effectivity }=\left(\left({C}_{\text{init}}-{C}_{\text{sed}}\right)/{C}_{\text{init}}\right)\times 100\%$$

Microsoft Excel (Office 2013) and GraphPad Prism (Version 10.0.0, GraphPad Software Inc., Boston, MA, USA) were used for the data evaluation and statistical tests. For the comparison with previous studies, a one-way ANOVA followed by a Holm–Sídák multiple comparison test was conducted. A one-way ANOVA followed by a Kruskal–Wallis test was conducted to compare the fresh TWW quality with the sedimentation-treated TWW quality. Limits of detection (LOD) are given in Supplementary Information B, Table B2. Concentrations below the LOD were replaced by half of the LOD.

### Evaluation of ecotoxicological risk

Fresh and sedimentation-treated TWW samples were classified into five classes, applying threshold concentrations for metals and PAHs for coastal waters, as given by the Norwegian Environmental Agency (Miljødirektoratet 2020). Generally, thresholds are less stringent for coastal waters as recipients than for freshwaters. Therefore, in this study, thresholds for coastal waters were chosen since most of the tunnels investigated finally discharge the TWW into a coastal water body.

## Results and discussion

### Fresh TWW quality

#### General characteristics

Table 2 presents the means, standard deviations, minimums, maximums, and median of the water quality parameters of the fresh TWW sampled from the sedimentation basins shortly after a wash. It can clearly be seen that the results vary considerably between the samples. The large variation might partly be due to inconsistent sampling locations and procedures due to the situations on-site, as described above. Also, the time between washing and sampling was not equal for all samplings. As big particles settle faster than small particles of the same density, later sampling could have been a reason for lower solid substance concentrations. Nevertheless, a correlation was found between the TS and the TSS. In a previous study, where TWW from 34 tunnels in Norway was investigated (Meland and Rødland 2018), pH (7.8 ± 0.4) was comparable to those in the current study (Table 2). The mean EC and the DOC were in the same range as those previously reported by Meland and Rødland (2018) (EC: 2453 ± 2474 µS/cm, DOC: 49 ± 44 mg/L, respectively). Consistent EC data were available for BjT, and the results support the seasonal trend observed in this study for sodium and chloride in the other tunnels (data not shown). The high concentrations observed corresponded with the wintertime and the application of road salt as de-icing agent. Seasonal trends for the other single water quality parameters could not be identified.

**Table 2:** Water quality parameters in the fresh tunnel wash water

	pH	EC	TUR	TSS	TS	DOC
		(µS/cm)	(NTU)	(mg/L)	(mg/L)	(mg/L)
Mean	8	1448	257	158	1112	23
SD (n)	0.48 (14)	798 (11)	287 (15)	169 (14)	441 (10)	18 (15)
CV	6.0%	55%	112%	107%	40%	77%
Minimum	7.3	82	0.57	1.7	538	2.9
Median	7.9	1475	145	83	1097	21
Maximum	8.9	2773	766	500	1712	80
Identified outliers	None	3643	None	1200	7157	None

Outliers are not included in mean, SD, minimum, medium, and maximum

*n* number of samples, *SD* standard deviation, *CV* coefficient of variation

A strong linear correlation was found between TUR and TSS (Supplementary Information A, Section A2, Fig. A2A). This allows the use of TUR as a reference when checking the consistency of TSS measurements.

#### Metals

**Fig. 1:**
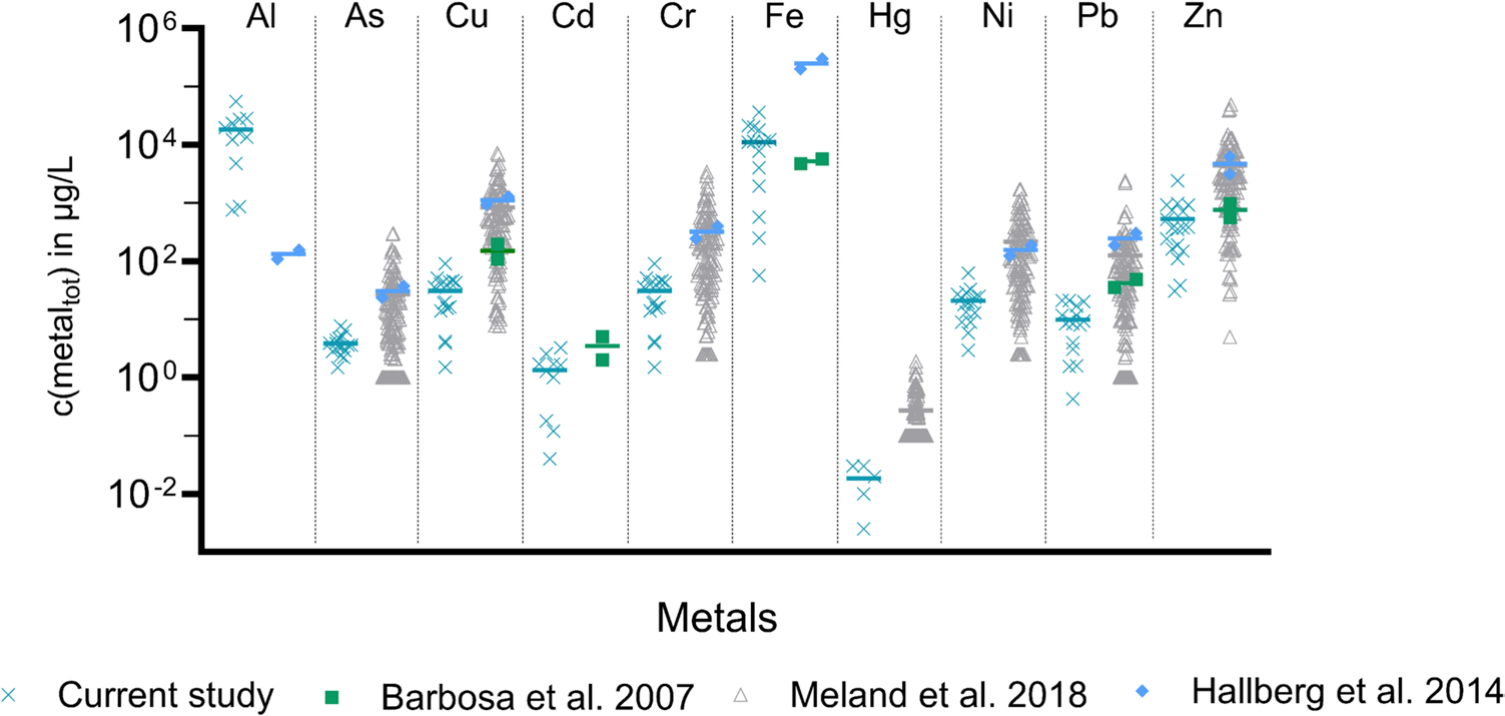
Total (particulate and dissolved) concentrations (*C*_init_) of metals found in TWW in this study (turquoise), compared to findings in previous studies. Horizontal bars represent means of individual findings in the respective study

Figure 1 compares the total metal concentrations (i.e. in the dissolved and particulate phases) with those found in previous studies (Supplementary Information B, Table B5). The test sites in these studies were different from those in this one. Total Al and total Fe were found in by far the highest mean concentrations with 18,349 and 11,147 µg/L, respectively, followed by total Zn with 533 µg/L and Mn with 365 µg/L. Compared with previous results from Hallberg et al. (2014), Al was found in a significantly higher mean concentration (*p* < 0.0001). The total concentrations for Fe were significantly lower (*p* < 0.001) than previously reported by Hallberg et al. (2014). Compared to the results of Meland and Rødland (2018), Zn was detected in significantly lower (*p* < 0.05) mean concentrations than previously reported, but not significantly different from those of Barbosa et al. (2007) and Hallberg et al. (2014) (*p* > 0.99). Nowadays, increasingly lighter, more impact- and heat-resistant materials, and material mixtures, are used in vehicles (Lipman and Maier 2021; Spreafico 2021). As Al fulfils a number of these properties, it is increasingly used in newer cars. In the cities of Oslo and Trondheim, the number of first registered cars, especially electrical vehicles, has increased in recent years (Statistisk sentralbyrå, 2023). In 2022, the share of first registrations of electric vehicles in Norway was as high as 76%, while registrations of gasoline (8.6%) and diesel (5.4%) vehicles fell to a low percentage (Statistisk sentralbyrå, 2023). The higher Al concentration observed in the current study could be explained by the trend towards the use of aluminium materials in newer vehicles and the higher number of newly registered vehicles in both cities. Total metal concentrations found for As (3.9 µg/L), Cu (113 µg/L), Cd (1.3 µg/L), Cr (31 µg/L), Hg (0.02 µg/L), Ni (21 µg/L), and Pb (9.8 µg/L) were not significantly different (*p* > 0.05) from those in previous findings (Barbosa et al. 2007; Hallberg et al. 2014; Meland and Rødland 2018).

**Fig. 2:**
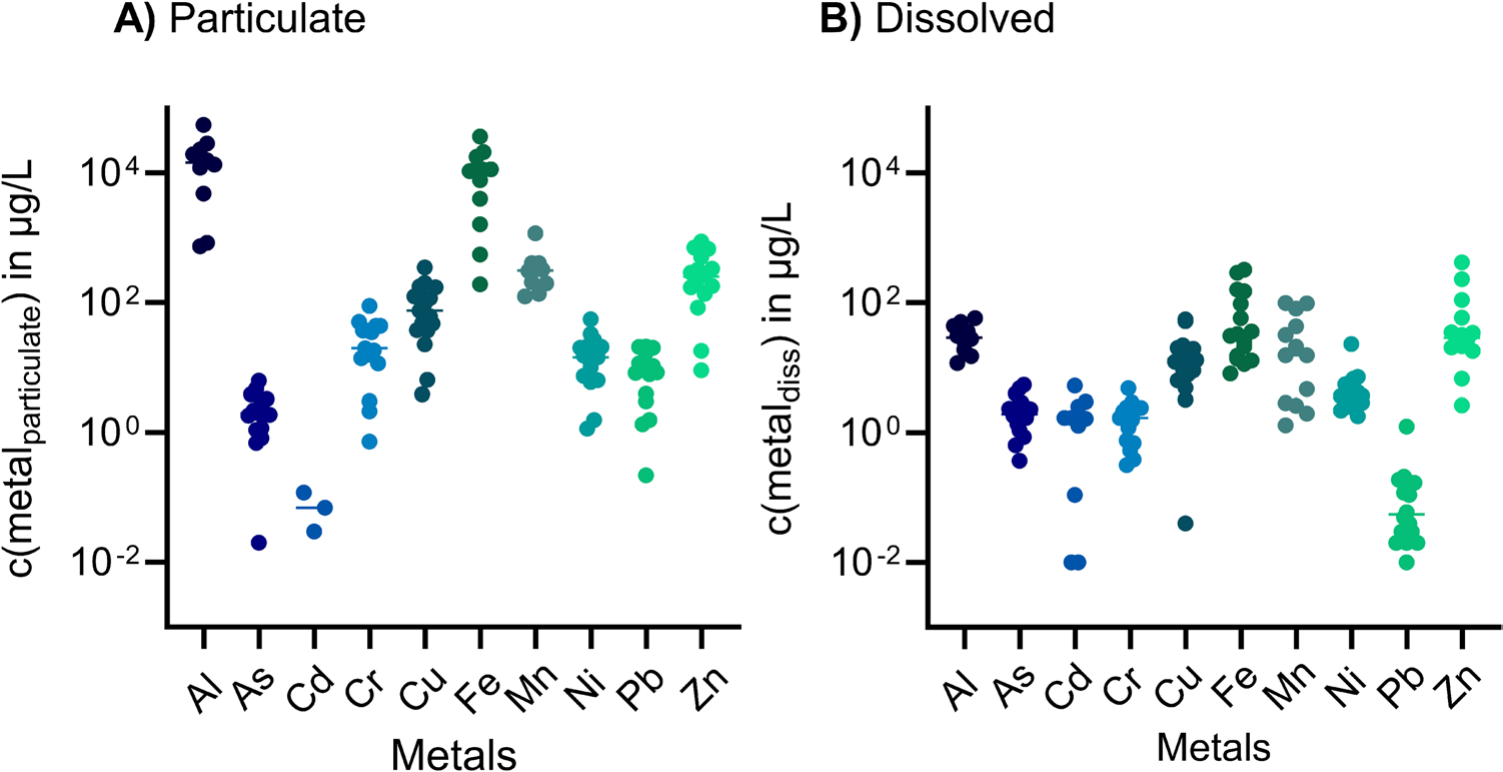
Metal concentrations in the particulate (**A**) and dissolved (**B**) fractions. Horizontal bars represent averages

Table 3 summarizes the concentrations of particulate and dissolved metals in fresh TWW. The total concentrations of Fe and Al exceeded the concentrations of any other metals by far. Most of the metals (i.e. Al, Cr, Cu, Fe, Mn, Pb, Zn) are primarily speciated in the particulate fraction, while more than 90% of the Cd and Ni are found in the dissolved form and approximately half of the As is in particulate or dissolved form. The data are visualized in Fig. 2. In the particulate fraction, the highest metal concentrations were observed for Al, Fe, Zn, and Mn. Particulate As, Pb, Ni, Cr, and Cu, however, were found in far lower concentrations, in the range of 2.3 to 99 µg/L. Cd was detected with a mean concentration of 0.07 µg/L. For the dissolved fraction, Zn, Fe, Al, and Mn were detected with the three highest concentrations.

**Table 3:** Concentrations (*C*_init_) of metals in the particulate and dissolved fractions in the fresh TWW

	**Particulate fraction (µg/L)**										**Dissolved fraction (µg/L)**									
	Al	As	Cd	Cr	Cu	Fe	Mn	Ni	Pb	Zn	Al	As	Cd	Cr	Cu	Fe	Mn	Ni	Pb	Zn
**Mean**	**17,447**	**2.3**	**0.073**	**28**	**99**	**11,226**	**362**	**17**	**9.7**	**310**	**32**	**2.2**	**1.7**	**1.7**	**16**	**80**	**32**	**5.1**	**0.16**	**68**
SD (*n*)	16,236 (10)	1.7 (15)	0.045 (3)	24 (15)	88 (17)	10,281 (12)	305 (10)	14 (15)	7.2 (15)	255 (16)	15 (10)	1.4 (17)	1.6 (10)	1.2 (17)	15 (18)	101 (16)	37 (13)	4.9 (17)	0.3 (16)	109 (16)
CV	93%	72%	61%	84%	89%	92%	84%	84%	74%	82%	48%	63%	94%	68%	90%	125%	114%	96%	191%	159%
Min	737	0.02	0.03	0.73	3.9	192	125	1.2	0.22	9	12	0.37	0.01	0.32	0.04	8.1	1.3	1.8	0.01	2.7
Median	14,631	2	0.07	20	75	10,997	315	14	8.4	253	29	2	1.7	1.7	13	32	16	3.7	0.055	28
Max	55,543	6.3	0.12	89	350	36,317	1181	56	21	871	58	5.4	5.3	4.8	55	325	99	23	1.2	419
Identified outliers	None	None	None	None	None	None	None	None	None	None	None	None	None	None	None	None	None	None	None	800
% in part. or diss. fraction	99.8	51.1	4.1	94.2	86.1	99.3	91.9	4.5	98.4	82.0	0.2	48.9	95.9	5.8	13.9	0.7	8.1	95.5	1.6	18.0

Outliers are not included in mean, SD, minimum, medium, and maximum

*n* number of samples, *SD* standard deviation, *CV* coefficient of variation

**Fig. 3:**
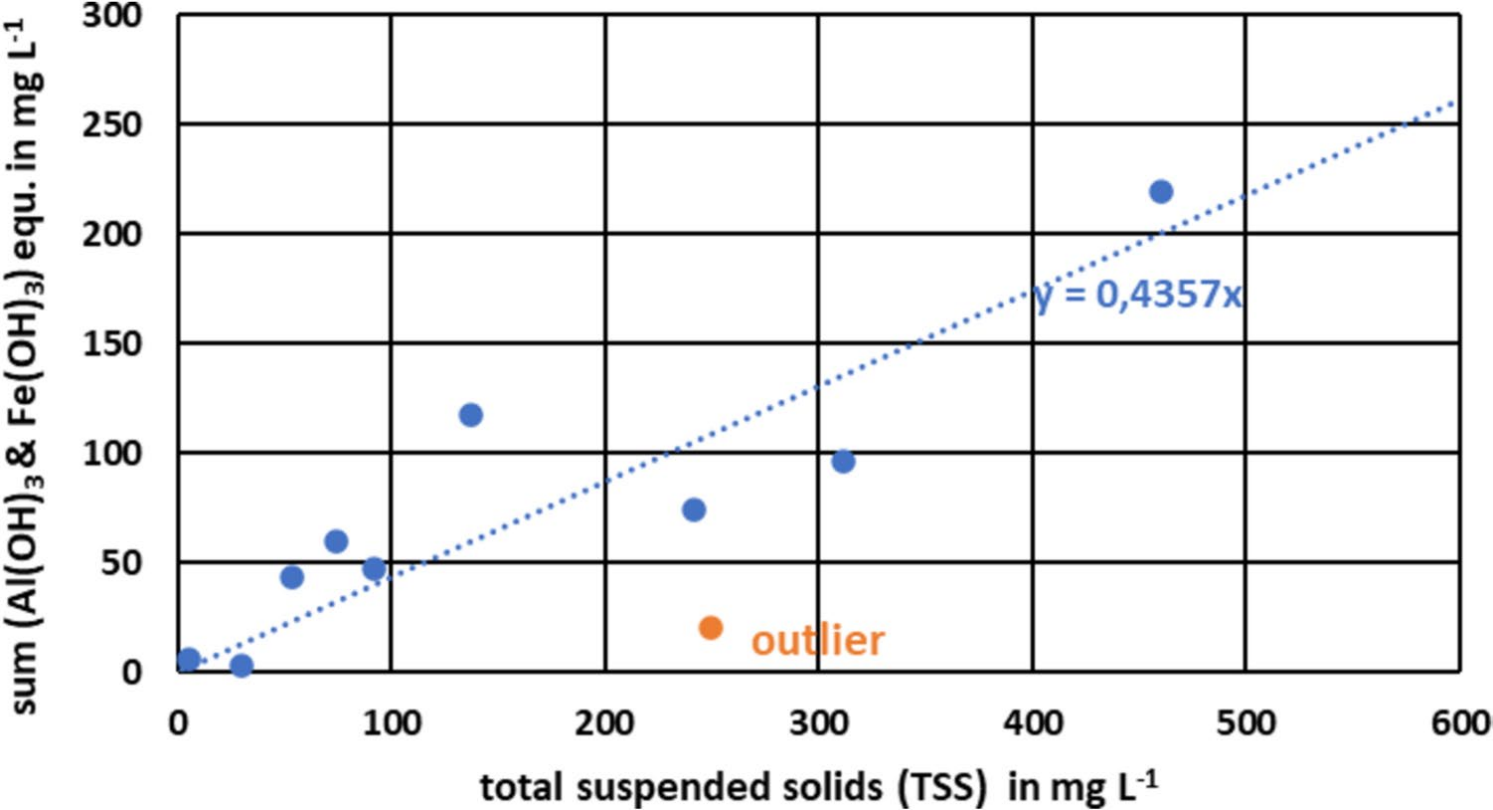
Concentration of iron and aluminium hydroxide equivalents versus total suspended solids in fresh TWW. The dashed line shows a linear regression while omitting the outlier

As the TWW was typically oxic, and considering the washing with high pressure, Fe will be present in its oxidized, three-valent form. At a pH of 8.0 ± 0.5 in the TWW (Table 2), the solubility of three-valent Fe and Al is very low. Both ions are then precipitated as Fe(OH)_3_ and Al(OH)_3_. (see e.g. Stumm and Morgan (1996)). It is therefore straightforward to convert the mass concentration of Fe and Al to iron and aluminium hydroxide equivalents and to compare their sum with the TSS concentration, as presented in Fig. 3. The regression shows that iron and aluminium hydroxide equivalents explain about 44% of the TSS in the fresh TWW.

It must be noted that the dissolved Fe concentrations of on average 80 µg/L (Table 3) were above the solubility of three-valent Fe, which is approximately 6 ng/L Fe. Complexation with organic matter and high ionic strength, as given in the TWW, is known to promote Fe mobilization (Chapman et al. 1998; Knocke et al. 1992; Liu and Millero 2002). For dissolved Al, however, the average concentration of 32 µg/L is in the order of magnitude of Al solubility which is approximately 15 µg/L at the respective pH. Zn and Cu also form insoluble hydroxides in water. At a pH of around 8, the solubility of Zn(II) in water is approximately 1 mg/L, while on average, about 100 µg/L dissolved and 400 µg/L particulate zinc were found. For Cu(II), the solubility is about 100 µg/L, while 17 µg/L dissolved and 100 µg/L particulate Cu were found on average in the fresh TWW samples. MnO_2_ is insoluble in water. In the TWW, it was found at on average 30 µg/L in the dissolved form, which might be due to complexation and colloidal Mn particles smaller than 0.45 µm. However, the concentration of particulate Mn was far higher, with on average 350 µg/L, compared to 30 µg/L in the dissolved phase.

Generally, the data reveal that metals are mainly found in the particulate form in the TWW if the total concentration is, at the respective pH, clearly higher than the solubility in the hydroxide or oxide form. In the TWW investigated, this was the case for Fe, Al, and Mn. Total Cu and Zn were on the edge or below their respective solubility.

#### PAHs

The concentrations of the Σ_16_PAH and the six selected PAHs in the particulate and dissolved fractions are summarized in Table 4. The data are visualized as a function of TSS in Supplementary Information A, Section A2, Fig. A3C + D. For particle-bound Σ_16_PAH, the average concentration in the particulate phase was 2.3 µg/L, and in the dissolved phase, it was 2.9 µg/L.

**Table 4:** Concentrations (*C*_init_) of the most abundant polycyclic aromatic hydrocarbons (PAHs) and the sum of EPA 16 PAHs (Σ_16_PAH) in the particulate and dissolved fractions in the fresh tunnel wash water

	**Particulate fraction (µg/L)**							**Dissolved fraction (µg/L)**						
	NAP	FLU	FLT	PHE	PYR	CHR	ΣPAH 16	NAP	FLU	FLT	PHE	PYR	CHR	ΣPAH 16
Mean	**1.6**	**0.12**	**0.077**	**0.021**	**0.095**	**0.068**	**2.3**	**0.25**	**0.06**	**0.19**	**0.23**	**0.27**	**0.17**	**2.9**
SD (*n*)	2.5 (9)	0.14 (9)	0.097 (9)	0.042 (9)	0.096 (9)	0.084 (9)	2.5 (9)	0.42 (13)	0.18 (15)	0.29 (15)	0.26 (15)	0.46 (15)	0.3 (15)	4.6 (15)
CV	154%	118%	127%	198%	101%	123%	107%	170%	294%	150%	114%	168%	179%	158%
Min	0.00003	0.003	0.0003	0.0003	0.0003	0.006	0.42	0.01	0.003	0.0003	0.003	0.003	0.006	0.097
Median	0.01	0.005	0.046	0.003	0.093	0.025	1.2	0.06	0.003	0.04	0.15	0.027	0.015	1.2
Max	6.8	0.31	0.3	0.13	0.29	0.25	7.4	1.5	0.69	0.94	0.94	1.2	0.84	18
Identified outliers	None	None	None	None	None	None	None	5.28 13.6	None	None	None	None	None	None
% in particulate or dissolved fraction	86.5	66.7	28.8	8.4	26.0	28.6	44.2	13.5	33.3	71.2	91.6	74.0	71.4	55.8

Outliers are not included in mean, SD, minimum, medium, and maximum

*n* number of samples, *SD* standard deviation, *CV* coefficient of variation

**Fig. 4:**
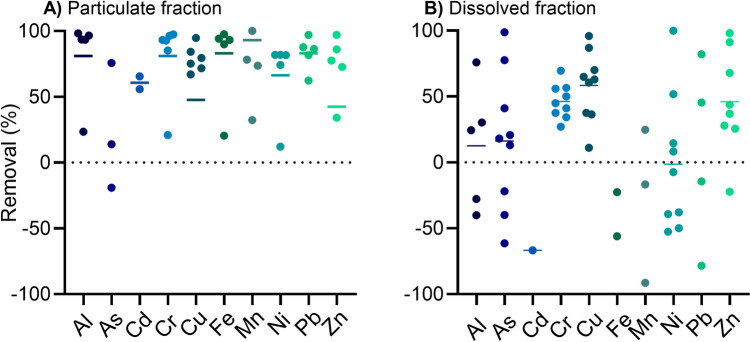
Removal effectivity of on-site sedimentation for metals in (**A)** the particulate fraction (*n* = 2–7) and (**B)** the dissolved fraction (*n* = 1–9). Means are presented as horizontal lines

Total particle-bound Σ_16_PAH concentrations ranged between 0.42 and 7.4 µg/L (Fig. 4 and Table 4). Allan et al. (2016) and Meland (2012a) previously reported comparable concentrations of 3.1–6.7 µg/g in sediments and particles from tunnels. For the dissolved phase, Paruch and Roseth (2008a) detected dissolved Σ_16_PAH concentrations of 2.9 µg/L in TWW from the Hanekleiv tunnel in Oslo, which is comparable to the average concentration of 2.9 µg/L in the dissolved phase found in our study. Paruch and Roseth (2008a) identified NAP as the main contributor with a concentration of 1.2 µg/L, followed by PHE (0.2 µg/L), PYR (0.6 µg/L), and FLT (0.4 µg/L) in their study, which is comparable to our observations (Table 4).

Overall, for 11 out of 16 PAHs, higher mean concentrations were observed in the dissolved fraction than in the particulate fraction in the fresh TWW (Supplementary Information B, Table B3). The concentrations of Σ_16_PAH found in the particulate phase and dissolved phase, respectively, were not significantly different (Table 4). For the particulate phase, a weak correlation with the concentration of TSS is suspected, but it cannot be proven as significant due to the scatter and limited number of data available.

### Sedimentation-treated TWW quality

#### General characteristics

For the general water quality parameters pH, EC, TS, and DOC, statistically significant differences to fresh TWW could not be confirmed. Due to the sedimentation process, TUR decreased to 20 NTU, and the TSS concentration to 15 mg/L (Table 5). All data are given in detail in Supplementary Information B, Table B4. However, TUR and TSS in the sedimentation-treated TWW samples were still high, indicating high particle concentrations.

**Table 5:** Water quality parameters in the sedimented tunnel wash water

	pH	EC (µS/cm)	TUR (NTU)	TSS (mg/L)	TS (mg/L)	DOC (mg/L)
Mean	7.6	1564	20	15	1234	15
SD (*n*)	0.5 (32)	970 (12)	18 (14)	20 (32)	523 (6)	3.9 (12)
CV	6.5%	62%	91%	128%	42%	25%
Minimum	5.4	52	2.3	1	704	10
Median	7.6	1512	20	7	1201	16
Maximum	8.6	3510	74	83	2134	22
Identified outliers	None	12,220	None	None	6911	None

Outliers are not included in mean, SD, minimum, medium, and maximum

*n* number of samples, *SD* standard deviation, *CV* coefficient of variation

#### Metals

Metal concentrations found in the sedimentation-treated TWW are summarized in Table 6. As for fresh TWW, in the particulate fraction, Fe (421 µg/L), Al (477 µg/L), Mn (79 µg/L), and Zn (79 µg/L) showed the highest mean concentrations. Except for Cd (0.03 µg/L), which had been measured at a very low mean concentration of 0.07 µg/L in the fresh TWW, for all other metals, the particulate fraction of sedimented TWW contained lower concentrations of metals than the fresh TWW.

**Table 6:** Concentrations (*C*_sed_) of metals in the particulate and dissolved fractions in the sedimented tunnel wash water

	**Particulate fraction (µg/L)**										**Dissolved fraction (µg/L)**									
	Al	As	Cd	Cr	Cu	Fe	Mn	Ni	Pb	Zn	Al	As	Cd	Cr	Cu	Fe	Mn	Ni	Pb	Zn
Mean	**477**	**1.5**	**0.03**	**1.4**	**25**	**421**	**79**	**5**	**0.79**	**79**	**24**	**3.1**	**0.014**	**1.3**	**9**	**137**	**69**	**3.6**	**0.25**	**32**
SD (*n*)	315 (6)	1.7 (5)	0.028 (2)	1 (8)	20 (9)	268 (7)	89 (8)	7.8 (8)	0.57 (7)	71 (8)	7.8 (7)	2.9 (13)	0.014 (3)	0.61 (13)	11 (13)	114 (12)	58 (11)	1.8 (13)	0.33 (12)	57 (11)
CV	66%	111%	94%	70%	83%	64%	112%	156%	72%	90%	32%	93%	103%	48%	127%	83%	85%	51%	133%	178%
Min	112	0.19	0.01	0.04	2	86	2.6	1	0.15	5	14	0.41	0.002	0.21	0.03	29	1.8	0.03	0.04	1.8
Median	420	0.66	0.03	1.6	17	427	44	2	0.59	48	25	2.6	0.01	1.4	4.6	97	47	3.1	0.14	18
Max	1041	4.1	0.05	2.7	66	822	224	24	1.5	193	36	12	0.03	2.4	43	419	164	7	1.2	203
Identified outliers	None	34	None	None	None	None	None	None	None	None	None	None	None	None	None	1000	1800	None	None	450
% in particulate or dissolved fraction	95.2	21.7	68.1	51.8	73.5	75.4	53.4	58.1	76.0	71.2	4.8	78.3	31.9	48.2	26.5	24.6	46.6	41.9	24.0	28.8

Outliers are not included in mean, SD, minimum, medium, and maximum

*n* number of samples, *SD* standard deviation, *CV* coefficient of variation

As for fresh TWW, the highest mean dissolved metal concentrations were found for Fe (137 µg/L), Al (24 µg/L), Mn (69 µg/L), and Zn (32 µg/L). Correlations between the TUR and TSS, as well as between iron and aluminium hydroxide equivalents and TSS, were identified (Supplementary Information A, Section A2, Figs. A2B and A3A).

#### PAHs

Selected PAH concentrations measured in sedimented TWW are summarized in Table 7. The Σ_16_PAH_dissolved_ concentrations ranged between 0.07 and 0.83 µg/L which is not statistically different from the fresh TWW concentration range (*p* > 0.05).

**Table 7:** Concentrations (*C*_sed_) of selected PAHs and the sum of EPA 16 PAHs (Σ_16_PAH) in the particulate and dissolved fractions in the sedimented tunnel wash water

	Particulate fraction (µg/L)					Dissolved fraction (µg/L)				
	NAP	FLU	PYR	CHR	ΣPAH 16	NAP	**PHE**	PYR	CHR	ΣPAH 16
Mean	0.97	0.011	0.025	0.072	1.2	0.022	0.0075	0.013	0.014	0.19
SD (*n*)	1.2 (4)	0.017 (5)	0.033 (5)	0.15 (5)	1.0 (5)	0.075 (25)	0.011 (28)	0.016 (28)	0.017 (28)	0.2 (28)
CV	119%	149%	130%	205%	84%	336%	152%	126%	119%	103%
Min	0.01	0.003	0.003	0.006	0.34	0.005	0.003	0.005	0.005	0.072
Median	0.72	0.003	0.01	0.006	0.86	0.006	0.005	0.005	0.006	0.1
Max	2.4	0.041	0.083	0.34	2.7	0.38	0.065	0.071	0.085	0.83
Identified outliers	36.96	None	None	None	37.96	9.5 29.6 9.6	None	None	None	None
% in particulate or dissolved fraction	97.8	59.5	65.8	83.7	86.3	2.2	40.5	34.2	16.3	13.7

Outliers are not included in mean, SD, minimum, medium, and maximum

*n* number of samples, *SD* standard deviation, *CV* coefficient of variation

Only five samples were analyzed for PAHs in the particulate fraction. The range of Σ_16_PAH_particulate_ was from 0.34 to 2.7 µg/L, while a single measurement of 37.4 µg/L, which was due to a measurement of particulate NAP as 36.96 µg/L, was identified as an outlier and not considered. Details are given in Supplementary Information B, Table B6. NAP, a small compound with two rings in its structure, is expected to be present in both the dissolved and particulate fraction. It has a tendency to adsorb to particles (Jesus et al. 2022). However, it is also classified as very mobile (Neumann and Schlieber 2019). As for the fresh TWW, NAP was the main contributor to the Σ_16_PAH concentrations in both the dissolved and particulate fractions of the sedimented TWW samples. All other compounds contributed less than 30% to the total Σ_16_PAH concentration.

### Effect of on-site sedimentation

#### General observations

Correlated pairs of TWW samples (fresh and sedimented; *n* = 13; Supplementary Information B, Table B7) were available for samples from BaT, BjT, and GrT. Therefore, the overall removal effectivity was calculated and evaluated for these three tunnels.

Mean removal effectivities for TUR (84%) and TSS (95%) prove significant removal of particles by sedimentation. However, the residual turbidity is still high, even after 43 days of sedimentation. This indicates that the remaining particles might be very small or colloidal, or their density might be very close to the density of water. It must be pointed out that the samples of sedimented TWW were taken close to the surface and thus do not indicate the effectivity of the sedimentation treatment with respect to the complete volume of TWW. However, they indicate what can potentially be achieved for the complete volume if the sedimentation is given enough time.

#### Metals

For the particulate fraction of metals, mean removal effectivity of > 80% for Mn (93%), Fe (83%), Pb (83%), Al (81%), and Cr (81%) was achieved through sedimentation (Fig. 4A). Ni (66%), Cd (61%), Cu (48%), and Zn (42%) were removed by sedimentation as well. Only As in the particulate phase could not be removed, more likely adsorption processes occurred and could explain the higher concentrations in the particulate phase after sedimentation.

For all metals analyzed for the dissolved fraction, the mean removal was below 60% (Al: 13%, As: 16%, Cr: 46%, Cu: 58%, Zn: 46%) or was not significant (Fig. 4B). Negative mean removal was observed for the metals Cd, Fe, Mn, Ni, and Pb. This could imply release from particles during sedimentation. Several studies on stormwater and road runoff indicate that the trace metals Al, Cu, Cr, Cd Fe, Pb, and Zn tend to occur in the particulate fraction and would explain the high removal effectivities of the particulate metal fraction through sedimentation (Baum et al. 2021; Huber et al. 2016; Muthukrishnan 2005). On the other hand, Ni and Mn, but also partly Cd, Cu, and Zn, are not significantly removed by sedimentation and remain in the dissolved phase (Baum et al. 2021; Huber et al. 2016; Muthukrishnan 2005). Baum et al. (2021) and Wang et al. (2020) investigated the distribution of particulate-bound metal concentrations across different particle size fractions in stormwater runoff and sediment deposited along roads, respectively. Both studies found higher metal concentrations of Cr, Cu, Cd, Ni, Pb, and Zn in the smaller fractions due to a higher organic content and particles attached to it. Although Zn is assumed to be present mainly in dissolved form, the particles adhering to the road sediments had a very high Zn content compared to the road sediments themselves. The smaller the individual fractions, the more mobile and the more difficult it is to remove them by sedimentation alone. Therefore, pollutants such as metals that are bound to finer particles pose a potential threat to the environment.

#### PAHs

**Fig. 5:**
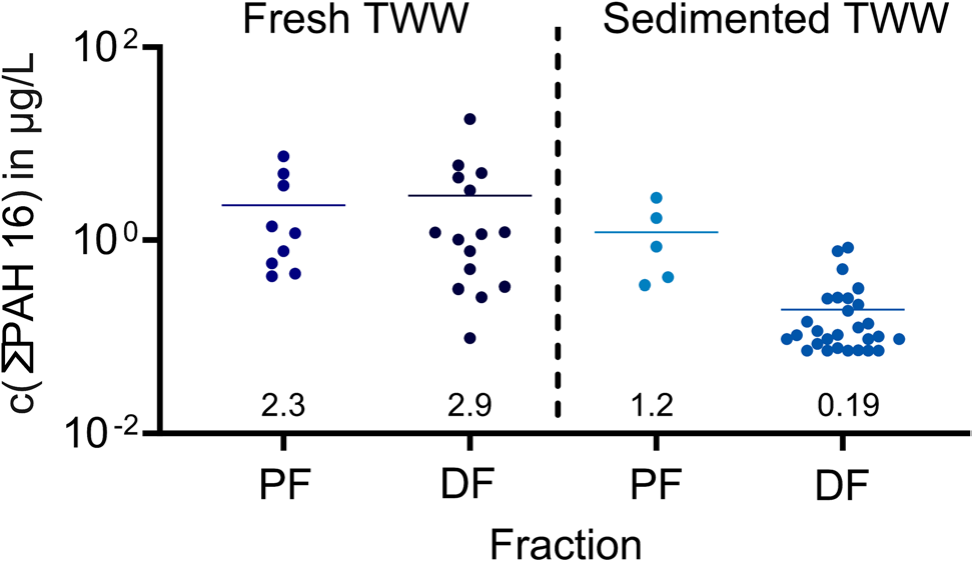
Sum of PAH16 polycyclic aromatic hydrocarbons (PAH16) concentrations in the particulate (PF) and dissolved (DF) fractions of the fresh (*n* = 9 (PF); *n* = 25(DF)) and sedimented (*n* = 5(PF); *n* = 29(DF)) TWW. Means are presented as a horizontal line and as a number

For Σ_16_PAH, concentrations in the particulate and dissolved fractions of fresh and sedimented TWW, respectively, are visualized in Fig. 5. The average concentration of Σ_16_PAH in the particulate fraction decreased from 2.3 to 1.2 µg/L, i.e. by 48%, while Σ_16_PAH in the dissolved fraction decreased from 2.9 to 0.2 µg/L, i.e. by 93%. It is important to realize that the removal of PAHs in the particulate fraction is far lower than the removal of TSS and TUR. Overall, as sedimentation mainly removes big particles, the results indicate that the majority of PAH16 in the particulate phase is bound to small particles, which remain in the TWW after sedimentation in the basin. Furthermore, the very high effectivity of removal of Σ_16_PAH from the dissolved phase indicates the adsorption of PAHs during the 35 days of residence between the tunnel wash and the sampling of sedimented TWW.

**Fig. 6:**
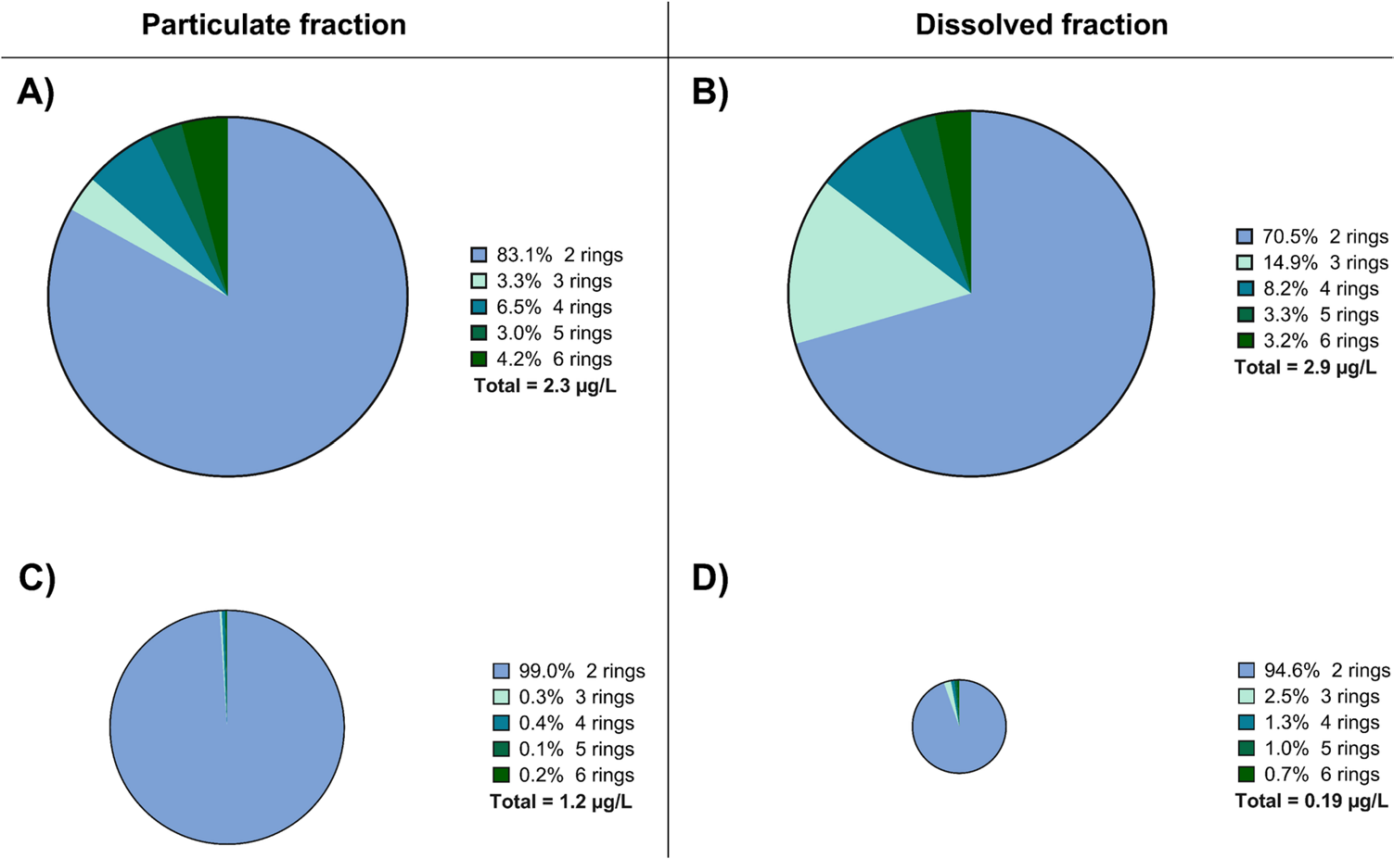
Distribution of polycyclic aromatic hydrocarbons (PAHs) in the particulate fraction for (**A)** fresh and (**C)** sedimented TWW and in the dissolved fraction for (**B)** fresh and (**D)** sedimented TWW based on their number of rings; *n* = 3–37. The areas of the circles are proportional to the average Σ_16_PAH concentrations

For further understanding, concentrations and removal effectivities for the single Σ_16_PAH compounds were evaluated in more detail. Figure 6 visualizes the concentrations of Σ_16_PAH in the fresh and sedimented fractions of TWW and the percentage contribution of PAHs with two to six rings, respectively. In all types of samples, compounds with two rings dominated. However, while they comprised about 83.1% and 70.5% (as mass concentrations) of the particulate and dissolved fractions of fresh TWW, respectively, their contribution increased to 99.0% and 94.6% in the sedimented TWW.

Positive removal effectivities for the particulate fraction were observed for the high molecular weight compounds FLT (96%), PYR (94%), CHR (74%), BbF (22%), BkF (37%), and BgP (82%), due to 35 days of sedimentation. In contrast, particulates NAP, ACY, ACE, FLU, PHE, ANT, and BaA, respectively, were not removed at all during the sedimentation process. In the dissolved fraction, removal effectivities of around 40% were observed for PYR (46%), CHR (41%), BbF (38%), DBA (42%), BgP (42%), and IND (40%). To a lower extent, the concentrations of ACE (23%), ANT (20%), FLT (34%), BaA (30%), BkF (13%), and BaP (22%) in the dissolved fraction decreased due to the sedimentation process.

It is interesting to realize that positive removal effectivities were mainly observed for the high molecular weight PAHs, and this accounts for both the particulate and dissolved fractions. These PAHs are known for their low solubility in water (Jesus et al. 2022). Consequently, it is likely that high molecular weight PAHs adsorb to particles and are finally removed from both the particulate and dissolved phases due to sedimentation. This distribution has previously been observed in freshwater systems (Rabodonirina et al. 2015), landfill leachates, and stormwater (Kalmykova et al. 2013). The low molecular weight compounds are less adsorbable and have previously been observed to occur as truly dissolved species in water (Kalmykova et al 2013).

In contrast, low molecular weight PAHs showed in many cases negative removal effectivities. This might indicate a release of PAHs from particles or other surfaces. Sabin et al. (2010) observed a release of PAHs from the sediments to the water column, containing mainly two- and three-ringed PAHs (low molecular weight compounds). In addition, degradation of higher molecular weight PAHs (four to six rings) during the sedimentation process might additionally contribute to an increase of low molecular weight PAH concentrations.

### Evaluation of the ecotoxicological risk

The Norwegian Environmental Agency (Miljødirektoratet) recommends threshold values for metal and organic pollutant concentrations for each recipient water body (coastal, fresh) as a tool to evaluate the ecotoxicological state. This classification is based on the environmental quality standards (EQS) for surface water set by the European Union (DIRECTIVE 2008/105/EC). In general, total concentrations in the whole water sample are considered, except for the metals Cd, Ni, and Pb, where dissolved concentrations are relevant. Furthermore, co-toxic effects, e.g. by metals and PAHs, Gauthier et al. (2014), are not considered in this classification scheme.

**Fig. 7:**
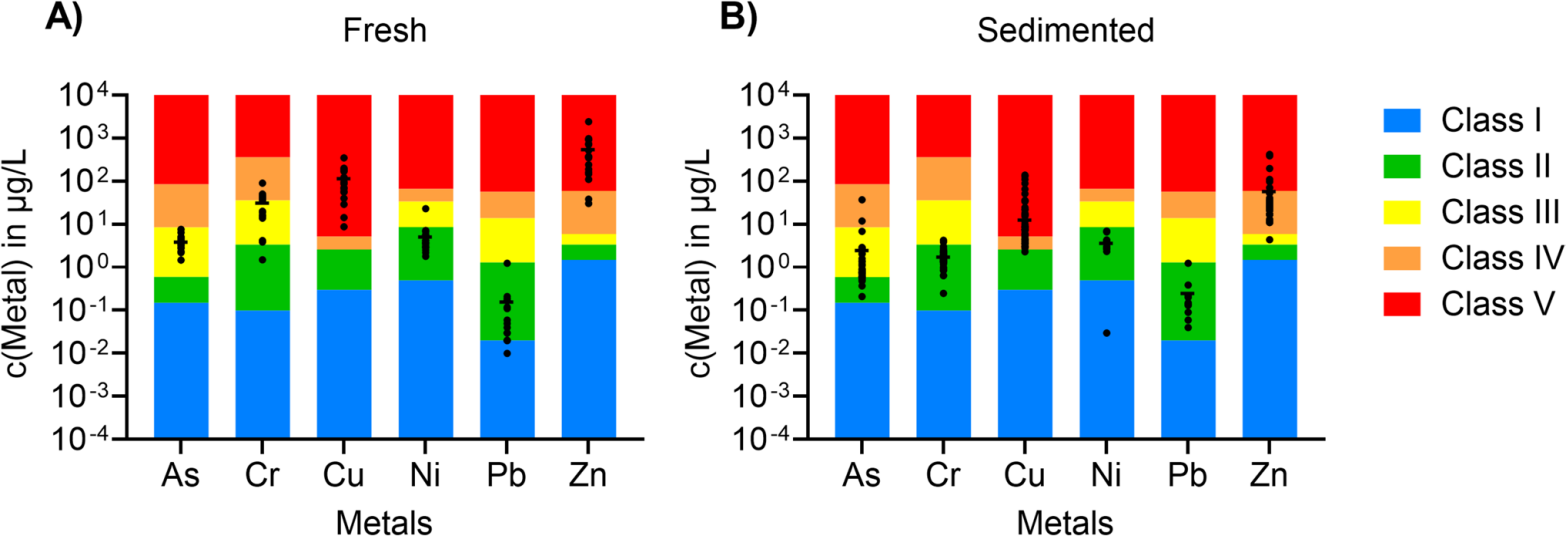
Comparison of the detected trace metal concentrations in (**A)** fresh tunnel wash water and (**B)** sedimented tunnel wash water for arsenic (As), cadmium (Cd), chromium (Cr), copper (Cu), nickel (Ni), lead (Pb), and zinc (Zn) with the environmental quality standards (Class I–V) for coastal water recipients. Horizontal black lines represent the average

For the fresh TWW, concentrations of three (Cd, Cu, Zn) out of eight metals resulted in a classification of poor water quality (Class IV and Class V) (Fig. 7A). Such concentrations cause acute toxic effects (Class IV) or lethal toxic effects (Class V) to organisms in the receiving water, even for short-term exposure. For the sedimented TWW, concentrations of two metals (Cu, Zn) were classified as poor water quality (Fig. 7B). Cadmium is not included in the figure since only a few measurements were available for that parameter.

**Fig. 8:**
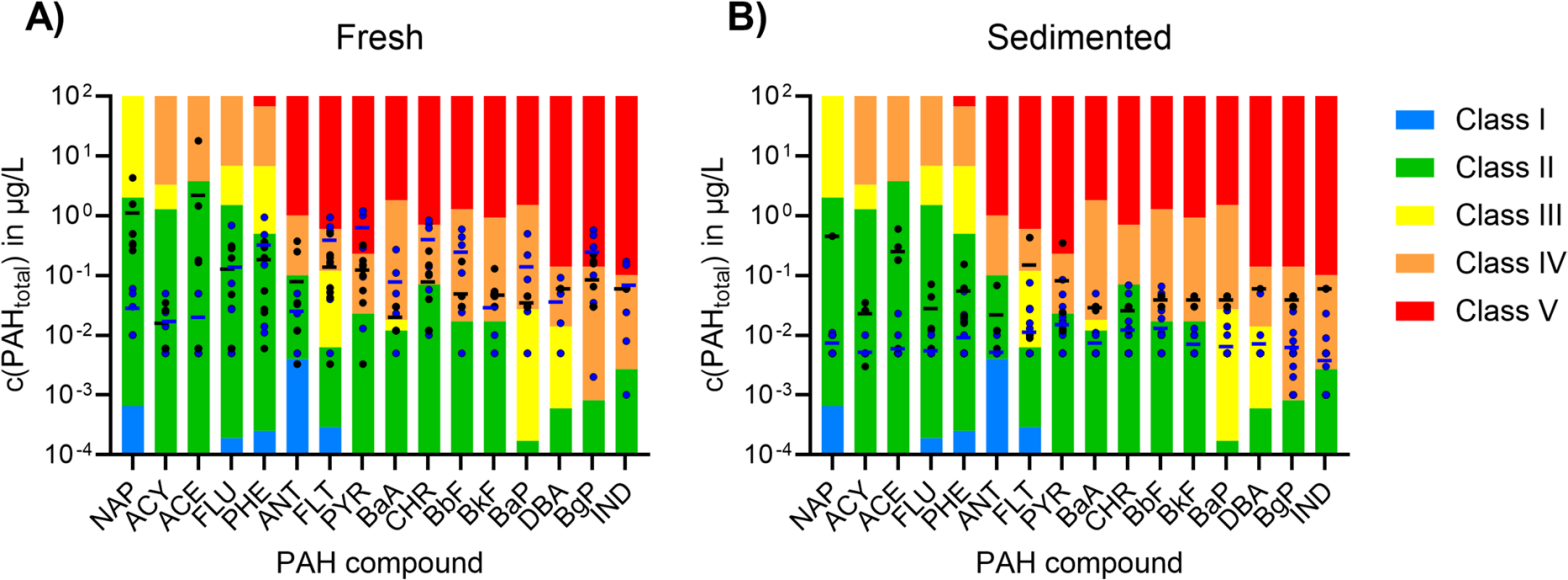
Comparison of the detected total (black) and dissolved (blue) concentrations in (**A)** fresh tunnel wash water and (**B)** sedimented tunnel wash water for the 16 EPA polycyclic aromatic hydrocarbons (PAHs) with the environmental quality standards (Class I–V) for coastal water recipients. The PAH compounds are listed in the order of increasing ring number; the number of rings is given in brackets after the compound’s abbreviation. Horizontal black and blue lines represent the average

Regarding PAHs in fresh TWW, 12 out of 16 PAHs indicated bad water quality (Class IV or more) in at least one sample (Fig. 8A). Some samples were only analyzed for dissolved PAHs, but due to their high concentration (up to Class V), they are presented. After sedimentation (Fig. 8B), for three PAHs, the classification had improved as they had been adsorbed to particles and (see the “PAHs” section) removed with particles. However, nine PAHs were still present in concentrations classified as bad quality (Class IV) in at least one sample. Generally, improvements due to sedimentation can be observed for all PAH compounds. This is in accordance with observations presented and discussed in the “PAHs” section.

### Impact on TWW management

Discharge of sedimented TWW is expected to cause environmental harm, and a secondary treatment step, like adsorption or nanofiltration, that aims at the removal of dissolved pollutants should be considered. It is not unreasonable to assume that more pollutants are present in TWW since more studies are available that have investigated pollutants originating from traffic (Ahmadireskety et al. 2022; Asheim et al. 2019; Vistnes et al. 2024), documenting the presence of per- and polyfluoroalkyl substances (PFAS), benzothiazoles (BTH), benzotriazoles (BTR), etc. In previous studies on road runoff and stormwater, filter media such as calcite, zeolite, iron filings, and biochar-added wood chips were found to effectively remove Cd, Cu, Pb, and Zn (Ashoori et al. 2019; Reddy et al. 2014). In addition, nature-based solutions such as constructed wetlands have shown promising removal effectivities for trace metals and PAHs in the treatment of road runoff and stormwater (Terzakis et al. 2008; Ventura et al. 2019). However, pollutant concentrations found in TWW are often higher than, for example, in road runoff, and many of them can be classified as being persistent, mobile, and toxic (Vistnes et al. 2024). This could point more towards technical treatment solutions for this point source pollution. Currently, separate treatments are often installed at each tunnel site. These are only operated for a fraction of their lifetime, depending on the wash frequency. Mobile solutions are a more efficient way since these can move from tunnel to tunnel and could be continuously operated.

## Summary and conclusions

The analysis of fresh and sedimented tunnel wash water (TWW) from 12 tunnels in Norway showed the following:

- TWW contains very high amounts of particulate matter, quantified as suspended solids (TSS) and turbidity (TUR). TUR and TSS correlate linearly.
- Concentrations of metals are generally far higher in the particulate fraction than in the dissolved fraction. Iron (Fe), aluminium (Al), manganese (Mn), and zinc (Zn) are dominating. Fe and Al precipitate as hydroxides and correspond to about 40% of the TSS. Particulate metals are effectively removed by sedimentation.
- Polycyclic aromatic hydrocarbon (PAH) concentration was higher in the dissolved fraction than in the particulate fraction. The particulate fraction decreased by about 50% due to sedimentation, a removal far lower than that of turbidity and TSS, indicating that PAH seems to be preferably adsorbed to very small particles that remain suspended even after 40 days of sedimentation. In contrast to metals, the concentration of PAHs in the dissolved fraction decreases by about 90% during sedimentation, most probably due to adsorption to particles that are subsequently removed. PAHs with three to six rings were preferably removed, since they may adsorb better to particles than PAHs with two rings.
- Ecotoxicological classification of Cu, Zn, and As resulted in a classification as poor or very poor water quality, and acute toxic effects can be expected after short-term exposition. This did not change after sedimentation, as the concentrations in the dissolved phase did not change. For PAHs, 8 out of 16 PAHs resulted in a classification as poor or very poor water quality for untreated TWW. This was the case for only two PAHs after sedimentation. This relates to the preferred removal of PAH with more rings, which are more harmful than compounds with fewer rings.
- Indications for a seasonal pollution variation have been seen, mainly related to the use of salt as a de-icing agent in road maintenance. However, this study was not designed to evaluate that specifically.
- Discharge of sedimented TWW is expected to cause environmental harm, and a secondary treatment step, which aims to remove dissolved pollutants poorly removed by gravity separation, should be considered. Infiltration solutions may be a suitable choice. However, pollutant concentrations can be higher in TWW than in road runoff, and more technical solutions for this point source should be considered. Today, separate treatment solutions are installed in each tunnel. These are only operated during a small fraction of the year. Mobile solutions could move from tunnel to tunnel and could be operated more efficiently.

## Supplementary Information

Below is the link to the electronic supplementary material.

Supplementary file1 (DOCX 3414 KB)

Supplementary file2 (XLSX 73 KB)

## Data Availability

All data that are supporting the results and analysis presented in this article can be found in the supplementary information, attached to the manuscript.

## Abbreviations

ADT: Annual average daily traffic
*C*_diss_: Concentration in the dissolved fraction
*C*_init_: Initial concentration
*C*_part_: Concentration in the particulate fraction
*C*_tot_: Total concentration, i.e. dissolved and particulate
*C*_sed_: Concentration after sedimentation
CV: Coefficient of variation
DF: Dissolved fraction
DOC: Dissolved organic carbon
EC: Electrical conductivity
EQS: Environmental quality standards
EU WFD: European Water Framework Directive
LOD: Limit of detection
*n*: Number of samples
PFAS: Per- and polyfluoroalkyl substances
SD: Standard deviation
SPE: Solid-phase extraction
SS: Suspended solids
TSS: Total suspended solids
TUR: Turbidity
TWW: Tunnel wash water

## Tunnels

BaT: Bamble (BaT)
BjT: Bjørnegård (BjT)
FlT: Fløyheia (FlT)
GaT: Granfoss (GaT)
GrT: Grillstad (GrT)
HhT: Hesthag (HhT)
NT: Nordby
SmT: Smestad
SrT: Strindheim
TbT: Torsbuåsen
TfT: Træfjell
TaT: Tåsen
**PAHs**: **Polycyclic aromatic hydrocarbons**
ACE: Acenaphthene
ACY: Acenaphthylene
ANT: Anthracene
BaA: Benzo[a]anthracene
BaP: Benzo[a]pyrene
BbF: Benzo[b]fluoranthene
BgP: Benzo[g,h,i]perylene
BkF: Benzo[k]fluoranthene
BTH: Benzothiazoles
BTR: Benzotriazoles
CHR: Chrysene
DBA: Dibenzo[a,h]anthracene
FLT: Fluoranthene
FLU: Fluorene
GC-MS: Gas chromatography-mass spectrometry
IND: Indeno[1.2.3-c.d]pyrene
NAP: Naphthalene
PHE: Phenanthrene
PYR: Pyrene
Σ_16_PAH_dissolved_: Total dissolved PAH concentrations
Σ_16_PAH_particulate_: Total particulate PAH concentrations

## References

[CR1] Ahmadireskety A, Da Silva BF, Robey NM, Douglas TE, Aufmuth J, Solo-Gabriele HM, Yost RA, Townsend TG, Bowden JA (2022) Per- and polyfluoroalkyl substances (PFAS) in street sweepings. Environ Sci Technol 56:6069–6077. 10.1021/acs.est.1c03766 34596397 10.1021/acs.est.1c03766

[CR2] Allan IJ, O’Connell SG, Meland S, Bæk K, Grung M, Anderson KA, Ranneklev SB (2016) PAH accessibility in particulate matter from road-impacted environments. Environ Sci Technol 50:7964–7972. 10.1021/acs.est.6b00504 27312518 10.1021/acs.est.6b00504 PMC5448791

[CR3] Asheim J, Vike-Jonas K, Gonzalez SV, Lierhagen S, Venkatraman V, Veivåg ILS, Snilsberg B, Flaten TP, Asimakopoulos AG (2019) Benzotriazoles, benzothiazoles and trace elements in an urban road setting in Trondheim, Norway: re-visiting the chemical markers of traffic pollution. Sci Total Environ 649:703–711. 10.1016/j.scitotenv.2018.08.299 30176481 10.1016/j.scitotenv.2018.08.299

[CR4] Ashoori N, Teixido M, Spahr S, LeFevre GH, Sedlak DL, Luthy RG (2019) Evaluation of pilot-scale biochar-amended woodchip bioreactors to remove nitrate, metals, and trace organic contaminants from urban stormwater runoff. Water Res 154:1–11. 10.1016/j.watres.2019.01.040 30763870 10.1016/j.watres.2019.01.040

[CR5] Barbosa AE, Saraiva J, Leitão T (2007) Evaluation of the runoff water quality from tunnel wash, in: Morrison, G.M., Rauch, S. (Eds.), Highway and urban environment: proceedings of the 8th Highway and Urban Environment Symposium. Springer, Dordrecht, pp. 345–358.

[CR6] Baum P, Kuch B, Dittmer U (2021) Adsorption of metals to particles in urban stormwater runoff—does size really matter? Water 13:309. 10.3390/w13030309

[CR7] Bellas J, Saco-Álvarez L, Nieto Ó, Beiras R (2008) Ecotoxicological evaluation of polycyclic aromatic hydrocarbons using marine invertebrate embryo – larval bioassays. Mar Pollut Bull 57:493–502. 10.1016/j.marpolbul.2008.02.039 18395228 10.1016/j.marpolbul.2008.02.039

[CR8] Borris M, Österlund H, Marsalek J, Viklander M (2016) Contribution of coarse particles from road surfaces to dissolved and particle-bound heavy metal loads in runoff: a laboratory leaching study with synthetic stormwater. Sci Total Environ 573:212–221. 10.1016/j.scitotenv.2016.08.062 27565530 10.1016/j.scitotenv.2016.08.062

[CR9] Chapman PM, Wang F, Janssen C, Persoone G, Allen HE (1998) Ecotoxicology of metals in aquatic sediments: binding and release, bioavailability, risk assessment, and remediation. Can J Fish Aquat Sci 55:2221–2243. 10.1139/f98-145

[CR10] Chen J, You L, Yang M, Wang X (2023) Traffic safety assessment and prediction under different lighting service states in road tunnels. Tunn Undergr Sp Technol 134:105001. 10.1016/j.tust.2023.105001

[CR11] Crabtree B, Dempsey P, Johnson I, Whitehead M (2008) The development of a risk-based approach to managing the ecological impact of pollutants in highway runoff. Water Sci Technol 57:1595–1600. 10.2166/wst.2008.269 18520017 10.2166/wst.2008.269

[CR13] Flanagan K, Branchu P, Boudahmane L, Caupos E, Demare D, Deshayes S, Dubois P, Meffray L, Partibane C, Saad M, Gromaire MC (2019) Retention and transport processes of particulate and dissolved micropollutants in stormwater biofilters treating road runoff. Sci Total Environ 656:1178–1190. 10.1016/j.scitotenv.2018.11.304 30625649 10.1016/j.scitotenv.2018.11.304

[CR14] Gauthier PT, Norwood WP, Prepas EE, Pyle GG (2014) Metal – PAH mixtures in the aquatic environment: a review of co-toxic mechanisms leading to more-than-additive outcomes. Aquat Toxicol 154:253–269. 10.1016/j.aquatox.2014.05.026 24929353 10.1016/j.aquatox.2014.05.026

[CR15] Hallberg M, Renman G, Byman L, Svenstam G, Norling M (2014) Treatment of tunnel wash water and implications for its disposal. Water Sci Technol 69:2029–2035. 10.2166/wst.2014.113 24845317 10.2166/wst.2014.113

[CR16] Hilliges R, Schriewer A, Helmreich B (2013) A three-stage treatment system for highly polluted urban road runoff. J Environ Manage 128:306–312. 10.1016/j.jenvman.2013.05.024 23770438 10.1016/j.jenvman.2013.05.024

[CR17] Huber M, Welker A, Helmreich B (2016) Critical review of heavy metal pollution of traffic area runoff: occurrence, influencing factors, and partitioning. Sci Total Environ 541:895–919. 10.1016/j.scitotenv.2015.09.033 26448594 10.1016/j.scitotenv.2015.09.033

[CR18] Jesus F, Pereira JL, Campos I, Santos M, Ré A, Keizer J, Nogueira A, Gonçalves FJM, Abrantes N, Serpa D (2022) A review on polycyclic aromatic hydrocarbons distribution in freshwater ecosystems and their toxicity to benthic fauna. Sci Total Environ 820:153282. 10.1016/j.scitotenv.2022.153282 35066033 10.1016/j.scitotenv.2022.153282

[CR19] Kalmykova Y, Björklund K, Strömvall AM, Blom L (2013) Partitioning of polycyclic aromatic hydrocarbons, alkylphenols, bisphenol A and phthalates in landfill leachates and stormwater. Water Res 47(3):1317–1328. 10.1016/J.WATRES.2012.11.054 23295068 10.1016/j.watres.2012.11.054

[CR20] Knocke WR, Conley L, van Benschoten JE (1992) Impact of dissolved organic carbon on the removal or iron during water treatment. Water Res 26:1515–1522. 10.1016/0043-1354(92)90072-C

[CR22] Laurén DJ, McDonald DG (1985) Effects of copper on branchial ionoregulation in the rainbow trout, *Salmo **gairdneri* Richardson - modulation by water hardness and pH. J Comp Physiol B 155:635–644. 10.1007/BF00694455

[CR23] Lipman TE, Maier P (2021) Advanced materials supply considerations for electric vehicle applications. MRS Bull 46:1164–1175. 10.1557/s43577-022-00263-z

[CR24] Liu X, Millero FJ (2002) The solubility of iron in seawater. Mar Chem 77:43–54. 10.1016/S0304-4203(01)00074-3

[CR26] Meland S (2012a) Kjemisk karakterisering av sediment fra Vassum sedimenteringsbasseng (No. 94) (in Norwegian: Chemical characterisation of sediment from Vassum sedimentation basin (No. 94)). Statens vegvesens, Oslo, Norway. vegvesen.brage.unit.no/vegvesen-xmlui/handle/11250/2508156?show=full.

[CR27] Meland S (2012b) Tunnelvaskevann – En kilde til vannforurensning (in Norwegian: Tunnel wash water - A source of water pollution). Vann 02:182–193

[CR28] Meland S, Borgstrøm R, Heier LS, Rosseland BO, Lindholm O, Salbu B (2010a) Chemical and ecological effects of contaminated tunnel wash water runoff to a small Norwegian stream. Sci Total Environ 408:4107–4117. 10.1016/j.scitotenv.2010.05.034 20547412 10.1016/j.scitotenv.2010.05.034

[CR29] Meland S, Heier LS, Salbu B, Tollefsen KE, Farmen E, Rosseland BO (2010b) Exposure of brown trout (*Salmo **trutta* L.) to tunnel wash water runoff - chemical characterisation and biological impact. Sci Total Environ 408:2646–2656. 10.1016/j.scitotenv.2010.03.025 20381128 10.1016/j.scitotenv.2010.03.025

[CR30] Meland S, Rødland ES (2018) Forurensning i tunnelvaskevann – en studie av 34 veitunneler i Norge (in Norwegian: Pollution in tunnel wash water - a study of 34 road tunnels in Norway). Vann 01:54–65

[CR31] Miljødirektoratet (2020) Grenseverdier for klassifisering av vann, sediment og biota (in Norwegian: Threshold values for classification of water, sediment and biota). Miljødirektoratet, Trondheim, Norway.

[CR32] Miljødirektoratet (2021) Vannportalen [WWW Document]. https://www.vannportalen.no/miljomal/miljomal2/

[CR33] Müller A, Österlund H, Marsalek J, Viklander M (2020) The pollution conveyed by urban runoff: a review of sources. Sci Total Environ 709:136125. 10.1016/j.scitotenv.2019.136125 31905584 10.1016/j.scitotenv.2019.136125

[CR34] Muthukrishnan S (2005) Treatment of heavy metals in stormwater runoff using wet pond, in: 21st Annual International Conference on Soils, Sediments and Water. United States Environmental Protection Agency, Amherst, p. 23.

[CR35] Neumann M, Schlieber I (2019) Protecting the sources of our drinking water: the criteria for identifying persistent, mobile and toxic (PMT) substances and very persistent and very mobile (vPvM) substances under EU Regulation REACH (EC) No 1907/2006. Umweltbundesamt, Dessau-Roßlau, Germany.

[CR36] Norwegian Public Roads Administration, 2014. Håndbok R610 Standard for drift og vedlikehold av riksveger (in Norwegian: Handbook R610 Standard for operation and maintenance of national roads).

[CR37] Paruch AM, Roseth R (2008a) Treatment of tunnel wash waters - experiments with organic sorbent materials. Part I: removal of polycyclic aromatic hydrocarbons and nonpolar oil. J Environ Sci 20:964–969. 10.1016/S1001-0742(08)62194-4 10.1016/s1001-0742(08)62194-4 18817076

[CR38] Paruch AM, Roseth R (2008b) Treatment of tunnel wash waters - experiments with organic sorbent materials. Part II: removal of toxic metals. J Environ Sci 20:1042–1045. 10.1016/S1001-0742(08)62147-6 10.1016/s1001-0742(08)62147-6 19143309

[CR39] Petersen K, Bæk K, Grung M, Meland S, Ranneklev SB (2016) In vivo and in vitro effects of tunnel wash water and traffic related contaminants on aquatic organisms. Chemosphere 164:363–371. 10.1016/j.chemosphere.2016.08.108 27596823 10.1016/j.chemosphere.2016.08.108

[CR40] Rabodonirina S, Net S, Ouddane B, Merhaby D, Dumoulin D, Popescu T, Ravelonandro P (2015) Distribution of persistent organic pollutants (PAHs, Me-PAHs, PCBs) in dissolved, particulate and sedimentary phases in freshwater systems. Environ Pollut 206:38–48. 10.1016/J.ENVPOL.2015.06.023 26142749 10.1016/j.envpol.2015.06.023

[CR41] Reddy KR, Xie T, Dastgheibi S (2014) Removal of heavy metals from urban stormwater runoff using different filter materials. J Environ Chem Eng 2:282–292. 10.1016/j.jece.2013.12.020

[CR42] Sabin LD, Maruya KA, Lao W, Diehl D, Tsukada D, Stolzenbach KD, Schiff KC (2010) Exchange of polycyclic aromatic hydrocarbons among the atmosphere, water, and sediment in coastal embayments of southern California. USA Environ Toxicol 29:265–274. 10.1002/etc.54 10.1002/etc.54 20821444

[CR44] Spreafico C (2021) Can modified components make cars greener? A life cycle assessment. J Clean Prod 307:127190. 10.1016/j.jclepro.2021.127190

[CR45] Srogi K (2007) Monitoring of environmental exposure to polycyclic aromatic hydrocarbons: a review. Environ Chem Lett 5:169–195. 10.1007/s10311-007-0095-0 29033701 10.1007/s10311-007-0095-0 PMC5614912

[CR46] Statens vegvesen (2022a) Vegbygging N200 [WWW Document] (in Norwegian: Road construction N200). https://viewers.vegnorm.vegvesen.no/product/859942?langUI=en&filePath=c1f0791d-b65e-4a2f-ad1d-dc31cbef3faa.pdf.

[CR47] Statens vegvesen (2022b) Vegkart [WWW Document] (in Norwegian: Road map). https://vegkart.atlas.vegvesen.no/#kartlag:geodata/@249152,6647677,14.

[CR48] Statens vegvesen (2022c) Vegnormal N500 Vegtunneler [WWW Document] (in Norwegian: Road standard N500 Road tunnels). https://viewers.vegnorm.vegvesen.no/product/859938?langUI=nb&filePath=afeb3fda-ec43-4cc1-9216-dca1e4f5642c.pdf.

[CR49] Statistisk sentralbyrå (2023) No title [WWW Document]. https://www.vegvesen.no/fag/fokusomrader/baerekraftig-mobilitet/nullutslippsmalene/.

[CR50] Stotz G, Holldorb C (2008) Highway tunnel washing and its effect on water quality, in: 11th International Converence on Urban Drainage, Edinburgh, Scotland, UK: 2008. pp. 1–9.

[CR51] Stumm W, Morgan JJ (1996) Aquatic chemistry: chemical equilibria and rates in natural waters, 3rd edn. Wiley, New York

[CR52] Terzakis S, Fountoulakis MS, Georgaki I, Albantakis D, Sabathianakis I, Karathanasis AD, Kalogerakis N, Manios T (2008) Constructed wetlands treating highway runoff in the central Mediterranean region. Chemosphere 72:141–149. 10.1016/j.chemosphere.2008.02.044 18396317 10.1016/j.chemosphere.2008.02.044

[CR53] The European Commission, 2004. Regulation EC No 648/2004/EC on detergents. Official Journal of the European Union 104, 1–54. https://eur-lex.europa.eu/legal-content/EN/TXT/PDF/?uri=CELEX:32004R0648&from=EN.

[CR54] Ventura D, Barbagallo S, Consoli S, Ferrante M, Milani M, Licciardello F, Cirelli GL (2019) On the performance of a pilot hybrid constructed wetland for stormwater recovery in Mediterranean climate. Water Sci Technol 79:1051–1059. 10.2166/wst.2019.103 31070585 10.2166/wst.2019.103

[CR55] Vistnes H, Sossalla NA, Røsvik A, Gonzalez SV, Zhang J, Meyn T, Asimakopoulos AG (2022) The determination of polycyclic aromatic hydrocarbons (PAHs) with PHLC-DAD-FLD and GC-MS techniques in the dissolved and particulate phase of road-tunnel wash water: a case study for cross-array comparisons and applications. Toxics 10:399. 10.3390/toxics10070399 35878304 10.3390/toxics10070399 PMC9321833

[CR56] Vistnes H, Sossalla NA, Asimakopoulos AG, Meyn T (2024) Occurrence of traffic related trace elements and organic micropollutants in tunnel wash water. Journal of Hazardous Materials. Vol. 465. 10.1016/j.jhazmat.2024.133498. 10.1016/j.jhazmat.2024.133498 38232556

[CR57] Wang Q, Zhang Q, Wang XC, Ge Y (2020) Size distributions and heavy metal pollution of urban road-deposited sediments (RDS) related to traffic types. Environ Sci Pollut Res 27:34199–34210. 10.1007/s11356-020-09653-9 10.1007/s11356-020-09653-9 32557049

