# Supplementary material for: Tunnel wash water in a cold climate: characteristics, ecotoxicological risk, and effect of sedimentation
Source: Environ Sci Pollut Res Int. 2025 Jan 6;32(5):2251–66. doi: 10.1007/s11356-024-35773-7 (PMC11802680; doi:10.1007/s11356-024-35773-7)
Supplement: Supplementary file 1 — Supplementary file1 (DOCX 3414 KB) [file 11356_2024_35773_MOESM1_ESM.docx]

**Electronic Supplementary Information**

**Tunnel wash water in a cold climate: characteristics, ecotoxicological risk, and effect of sedimentation**

Nadine A. Sossalla ^a^, Wolfgang Uhl ^b,c^, Hanne Vistnes ^a^, Subhash Srikantha Rathnaweera ^b^, Eilen Arctander Vik ^b^, Thomas Meyn ^a,^*

^a^ Norwegian University of Science and Technology, Department of Civil and Environmental Engineering, S. P. Andersens veg 5, 7031 Trondheim, Norway

^b^ Aquateam COWI AS, Karvesvingen 2, 0579 Oslo, Norway

^c^ COWI AS, Karvesvingen 2, 0579 Oslo, Norway

**Section A1. Supplementary description**

## Metal and polycyclic aromatic hydrocarbon analysis

A fractionation step was done prior metal analysis. Two subsamples of 15 mL each were extracted from each TWW sample: one was not filtered while the other one was filtered through a 0.45 µm filter. Then, samples were acidified with 65% nitric acid (HNO_3_) and stored at 4 ̊C until further analysis. The unfiltered samples were further microwave digested by UltraClave and diluted prior to analysis. Metal concentrations were analysed by inductively coupled plasma mass spectrometry (ICP-MS; Agilent 8800 ICP-QQQ).

A method for analysis of PAHs has been previously established by Vistnes et al. (2022). Briefly, frozen samples were thawed, and 50 mL sample was filtered with a glass microfiber filter (pore size 0.45 µm) to get a particulate fraction (> 0.45 µm) and a dissolved fraction (< 0.45 µm). The particulate fraction filters were freeze dried and extracted by ASE. The extract was evaporated down to 2 mL at 40 °C, added 10 mL ethyl acetate, filtered with a polyethersulfone (PES) filter with a 0.45 µm pore size, and reconcentrated to 1 mL with ethyl acetate (EtAc). The dissolved fraction was extracted by SPE (Bondesil C18 cartridges) conditioned with dichloromethane (DCM) and methanol and equilibrated with high-performance liquid chromatography grade water. The samples were extracted by gravity, then the cartridges were dried by centrifugation and the elution was performed by the addition of acetone and DCM. The extracts were evaporated down to 0.5 mL at 40 °C, 0.5 mL acetonitrile (ACN) was added, reconcentrated down to 0.5 mL, and diluted to 1 mL with ACN. 0.5 mL of the extract was then evaporated to dryness and diluted with EtAc to 0.5 mL. Both particulate and dissolved fraction extracts were stored at – 20 °C until analysis by GC-MS.

**Section A2. Supplementary graphs**

The correlation analysis identified one sample (BaT_F_220519) as an outlier for the suspended solids and was therefore excluded from further analysis.


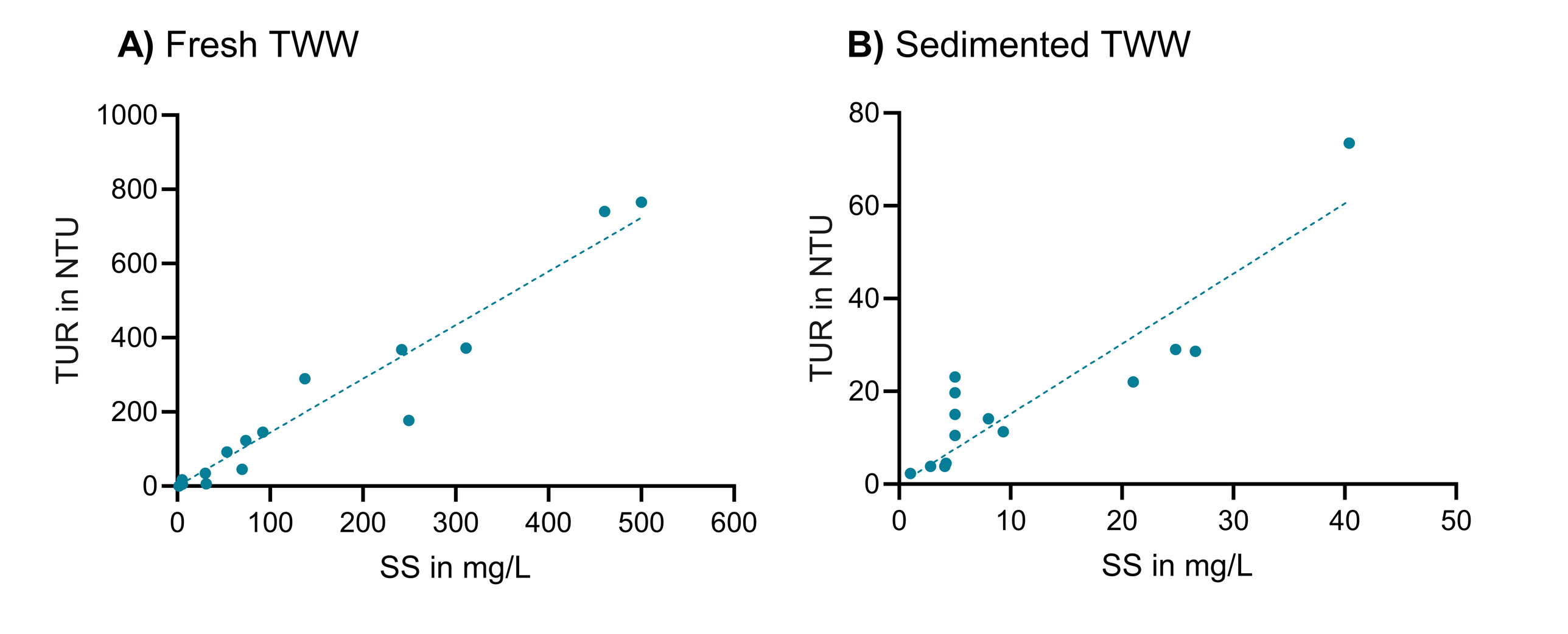


**Figure A2.** Turbidity (NTU) as a function of total suspended solids (TSS) in A) fresh tunnel wash water and B) sedimented tunnel wash water. The proposed correlation is given as dotted line.





**Figure A3.** The A) apparent iron hydroxide and aluminum hydroxide (∑Fe(OH)_3_+∑Al(OH)_3_), B) sum of the remaining studied metals (∑HMe), and the sum of the polycyclic aromatic hydrocarbons (∑PAH) in the C) particulate and D) dissolved fraction as a function of the suspended solids TSS in the sedimented tunnel wash water. The proposed correlation is given as dotted line.





**Figure A4.** Correlation analysis of iron (Fe), aluminum (Al), sum of the remaining trace metals (∑HMe), and the 16 EPA PAHs (∑PAH) in the dissolved fraction for the fresh (left column) and the sedimented (right column) tunnel wash water over the TSS.

**References**

Karlsen, S.E., 2021. Sofie Eivik Karlsen Master ’ s thesis Evolution of tunnel wash water quality during sedimentation.

Vistnes, H., Sossalla, N.A., Røsvik, A., Gonzalez, S. V., Zhang, J., Meyn, T., Asimakopoulos, A.G., 2022. The Determination of Polycyclic Aromatic Hydrocarbons (PAHs) with PHLC-DAD-FLD and GC-MS Techniques in the Dissolved and Particulate Phase of Road-Tunnel Wash Water: A Case Study for Cross-Array Comparisons and Applications. Toxics 10, 399. https://doi.org/10.3390/toxics10070399
